# The third of a series of articles for the 60^th^ anniversary of the Brazilian Society of Genetics

**DOI:** 10.1590/1678-4685-GMB-2017-00010001

**Published:** 2017-03-20

**Authors:** Francisco Mauro Salzano, Fabrício Rodrigues dos Santos, Klaus Hartfelder, Carlos F.M. Menck

**Affiliations:** 1 Departamento de Genética, Instituto de Biociências, Universidade Federal do Rio Grande do Sul, Porto Alegre, RS, Brazil.; 2 Departamento de Biologia Geral, Instituto de Ciências Biológicas, Universidade Federal de Minas Gerais, Belo Horizonte, MG, Brazil.; 3 Departamento de Biologia Celular e Molecular e Bioagentes Patogênicos, Faculdade de Medicina de Ribeirão Preto, Universidade de São Paulo, Ribeirão Preto, SP, Brazil.; 4 Departamento de Microbiologia, Instituto de Ciências Biomédicas, Universidade de São Paulo, São Paulo, SP, Brazil.

This issue of GMB (40.1) brings the third and last series of articles especially written and dedicated to the 60^th^ anniversary of the Brazilian Society of Genetics (SBG). This series includes two review and four research articles. In the first, Dr. Willy Beçak, former long-term Director of the Butantan Institute in São Paulo, and his group present an extensive overview on papillomaviruses in vertebrates, as important pathogenic agents for cancer. They direct special attention to bovine papillomaviruses, which are not only of eminent economic relevance, but also have great potential as experimental models for disease control. Dr. Roberto Giugliani and his colleagues from the Medical Genetics group of the Clinical Hospital in Porto Alegre, report on their experience as a Reference Center for lysosomal storage diseases in Brazil. They provide an overview on diagnostic methods and on frequency estimates for Brazil that should be of value for planning health care for these diseases. Dr. Samuel Goldenberg, current Director of Fiocruz Carlos Chagas Institute in Curitiba, and his group present an in-depth review on RNA binding proteins in single cell eukaryotes. They focus on a comparison between RBPs of *Trypanosoma cruzi* and those of *Sacharomyces cervisiae*, with the aim of inferring from this model organism on possible functions for these proteins in the Chagas disease causing pathogen *T. cruzi*. The next two research articles contributed by Dr. Fabrício Rodrigues dos Santos and colleagues are on the phylogeographic history and population genetics structure of two anteater species from Brazil. Anteaters are an emblematic group of phylogenetically ancient South American mammals, Xenarthra, subject to serious threat from changes in land use. For the smallest of these, the arboreal silky anteater, they focus on phylogeographic patterns of its populations and compare and relate these with environmental changes that occurred in the continent's recent history. For the giant anteater, which is on the red list of threatened species, they analyzed the genetic structure of its populations that become more and more isolated due to intensification of land use, especially in Central Brazil, with the aim of contributing to conservation management. The last article in this series comes from the group of Klaus Hartfelder, reporting on epigenetic marks in the stingless bee *Melipona scutellaris. Melipona* bees have long drawn attention due to their special genetic mechanism underlying caste determination in social insects, proposed over 50 years ago by Dr. Warwick E. Kerr. The data on global DNA methylation and levels of histone methylation and phosphorylation may now shed new light on the developmental genetics of these bees.

We hope you enjoy these high quality scientific articles and the other excellent works published in this issue, and we want to thank all authors who participated in these three series of 18 articles dedicated to the 60^th^ anniversary of SBG.

Fabrício Rodrigues dos Santos, Francisco Mauro Salzano, Carlos FM Menck and
Klaus Hartfelder FRS and FMS are Guest Editors of the SBG 60 years Special
Series of Articles and CFMM and KH Editors of Genetics and Molecular
Biology

